# Supplementary material for: Wild Plants Used as Herbs and Spices in Italy: An Ethnobotanical Review
Source: Plants (Basel). 2021 Mar 16;10(3):563. doi: 10.3390/plants10030563 (PMC8002413; doi:10.3390/plants10030563)
Supplement: Supplementary file 1 [file plants-10-00563-s001.pdf]

Supplementary

Papers which contain reports of wild plants specifically used for flavoring followed by the number of species from each source (in brackets and bold)

16. Idolo, M.; Motti, R.; and Mazzoleni, S. Ethnobotanical and Phytomedicinal knowledge in a long-history protected area, the Abruzzo, Lazio and Molise National Park (Italian Apennines). J. Ethnopharmacol. 2010, 127(2): 379–395. **(22)**

17. Vitalini, S.; Iriti, M.; Puricelli, C.; Ciuchi, D.; Segale, A.; Fico, G. Traditional knowledge on medicinal and food plants used in Val San Giacomo (Sondrio, Italy)—An alpine ethnobotanical study. J. Ethnopharmacol. 2013, 145(2), 517-529. **(5)**

21. Guarrera, P. M.; Salerno, G.; Caneva, G. Food, flavouring and feed plant traditions in the Tyrrhenian sector of Basilicata, Italy. J. Ethnobiol. Ethnomedicine 2006, 2(1), 37. **(5)**

22. Lentini, F.; Venza, F. Wild food plants of popular use in Sicily. J. Ethnobiol. Ethnomedicine 2007, 3(1), 15. **(17)**

23. Vitalini, S.; Puricelli, C.; Mikerezi, I.; Iriti, M. Plants, people and traditions: ethnobotanical survey in the Lombard Stelvio national park and neighbouring areas (Central Alps, Italy). J. Ethnopharmacol. 2015, 173, 435-458. **(17)**

24. Leporatti, M. L.; Guarrera, P. M. Ethnobotanical remarks in Capitanata and Salento areas (Puglia, southern Italy). Etnobiología 2007, 5(1), 51-64. **(1)**

33. Mattalia, G.; Corvo, P.; Pieroni, A. The virtues of being peripheral, recreational, and transnational: local wild food and medicinal plant knowledge in selected remote municipalities of Calabria, Southern Italy. Ethnobot. Res. Appl. 2020, 19, 1-20. **(13)**

34. Mattalia, G.; Sõukand, R.; Corvo, P.; Pieroni, A. Blended divergences: local food and medicinal plant uses among Arbëreshë, Occitans, and autochthonous Calabrians living in Calabria, Southern Italy. Plant Biosystems 2020, 154(5), 615-626. **(5)**

35. Maruca, G.; Spampinato, G.; Turiano, D.; Laghetti, G.; Musarella, C. M. Ethnobotanical notes about medicinal and useful plants of the Reventino Massif tradition (Calabria region, Southern Italy). Genet. Resour. Crop Evol. 2019, 66(5), 1027-1040. **(4)**

43. Uncini Manganelli, R. E.; Camangi, F.; Tomei, P. E.; Oggiano, N. L'uso delle erbe nella tradizione rurale della Toscana. Voll. I-II. ARSIA-Regione Toscana, Firenze. 2002. **(6)**

72. Mattalia, G.; Quave, C. L.; Pieroni, A. Traditional uses of wild food and medicinal plants among Brigasc, Kyé, and Provençal communities on the Western Italian Alps. Genet. Resour. Crop Evol. 2013, 60(2), 587-603. **(10)**

73. Motti, R.; Bonanomi, G.; Lanzotti, V.; Sacchi, R. The Contribution of Wild Edible Plants to the Mediterranean Diet: An Ethnobotanical Case Study Along the Coast of Campania (Southern Italy). Econ. Bot. 2020, 74(3), 249-272. **(9)**

74. Bellia, G.; Pieroni, A. Isolated, but transnational: the glocal nature of Waldensian ethnobotany, Western Alps, NW Italy. J. Ethnobiol. Ethnomedicine 2015, 11(1), 37. **(9)**

75. Sansanelli, S.; Tassoni, A. Wild food plants traditionally consumed in the area of Bologna (Emilia Romagna region, Italy). J. Ethnobiol. Ethnomedicine 2014, 10(1), 69. **(10)**

76. Pieroni, A.; Giusti, M. E. Alpine ethnobotany in Italy: traditional knowledge of gastronomic and medicinal plants among the Occitans of the upper Varaita valley, Piedmont. J. Ethnobiol. Ethnomedicine 2009, 5(1), 32. **(5)**

77. Pieroni, A. Gathered wild food plants in the upper valley of the Serchio River (Garfagnana), Central Italy. Econ. Bot. 1999, 53(3), 327-341. **(6)**

78. Coassini Lokar, L.; Poldini, L.; Angeloni Rossi, G. Appunti di etnobotanica del Friuli-Venezia Giulia. Gortania-Atti del Museo Friulano di Storia Naturale 1983, 4, 101-152. **(3)**

79. Signorini, M. A.; Piredda, M.; Bruschi, P. Plants and traditional knowledge: An ethnobotanical investigation on Monte Ortobene (Nuoro, Sardinia). J. Ethnobiol. Ethnomedicine 2009, 5(1), 6. **(6)**

80. Cornara, L.; La Rocca, A.; Marsili, S.; Mariotti, M. G.. Traditional uses of plants in the Eastern Riviera (Liguria, Italy). J. Ethnopharmacol. 2009, 125(1), 16-30. **(12)**

81. Savo, V.; Salomone, F.; Bartoli, F.; Caneva, G. When the local cuisine still incorporates wild food plants: the unknown traditions of the Monti Picentini Regional Park (Southern Italy). Econ. Bot. 2019, 73(1), 28-46. **(8)**

82. Montesano, V.; Negro, D.; Sarli, G.; De Lisi, A.; Laghett, I. G.; and Hammer, K. Notes about the uses of plants by one of the last healers in the Basilicata region (South Italy). J. Ethnobiol. Ethnomedicine 2012, 8: 15. **(1)**

83. Menale, B.; Amato, G.; Di Prisco, C.; Muoio, R. Traditional uses of plants in north-western Molise (Central Italy). Delpinoa 2006, 48, 29-36. **(3)**

84. Pieroni, A.; Nebel, S.; Quave, C.; Münz, H.; Heinrich, M. Ethnopharmacology of liakra: traditional weedy vegetables of the Arbëreshë of the Vulture area in southern Italy. J. Ethnopharmacol. 2002, 81(2), 165-185. **(7)**

85. Mautone, M.; L. De Martino, and V. De Feo. Ethnobotanical research in Cava de'Tirreni area, Southern Italy. about the uses of plants by one of the last healers in the Basilicata region (South Italy). J. Ethnobiol. Ethnomedicine 2019, 15(1):50. **(3)**

86. Motti, R. and P. Motti. An ethnobotanical survey of useful plants in the agro Nocerino Sarnese (Campania, southern Italy). Hum. Ecol. 2017, 45:865–878. **(4)**

87. Arcidiacono, S.; Costa, R.; Marletta, G.; Pavone, P.; Napoli, M. Usi popolari delle piante selvatiche nel territorio di Villarosa (EN–Sicilia Centrale). Quad. Bot. Amb. Appl. 2010, 1, 95-118. **(4)**

88. Guarrera, P. M. Le piante nelle tradizioni popolari della Sicilia. Erboristeria domani, 2009, 1, 46-55. **(2)**

89. Cornara, L.; La Rocca, A.; Terrizzano, L.; Dente, F.; Mariotti, M. G. Ethnobotanical and phytomedical knowledge in the North-Western Ligurian Alps. J. Ethnopharmacol. 2014, 155(1), 463-484. **(9)**

90. Salerno, G. P. M. Guarrera. Ricerche etnobotaniche nel Parco Nazionale del Cilento e Vallo di Diano: il territorio di Castel San Lorenzo (Campania, Salerno). Informatore Botanico Italiano 2008, 40:165–181. **(4)**

91. Arcidiacono, S.; Napoli, M.; Oddo, G.; Pavone, P. Piante selvatiche d'uso popolare nei territori di Alcara Li Fusi e Militello Rosmarino (Messina, NE Sicilia). Quad. Bot. Amb. App. 2007, 18, 105-146. **(5)**

92. Nebel, S.; Pieroni, A.; Heinrich, M. Ta chòrta: wild edible greens used in the Graecanic area in Calabria, Southern Italy. Appetite 2006, 47(3), 333-342. **(3)**

93. Motti, R.; V. Antignani, and M. Idolo. Traditional plant use in the Phlegraean fields Regional Park (Campania, southern Italy). Hum. Ecol. 2009, 37:775–782. **(2)**

94. Atzei, A. D.; Orioni, S.; Sotgiu, R. Contributo alla conoscenza degli usi etnobotanici nella Gallura (Sardegna). Boll. Soc. Sarda Sci. Nat. 1991, 28, 137-177. **(2)**

95. Di Novella, R.; N. Di Novella, L. De Martino, E. Mancini, and V. De Feo. Traditional plant use in the National Park of Cilento and Vallo di Diano, Campania, Southern Italy. J. Ethnopharmacol. 2013, 145:328–342. **(5)**
